# Supplementary material for: A timeline of bacterial and archaeal diversification in the ocean
Source: eLife. 2023 Dec 7;12:RP88268. doi: 10.7554/eLife.88268 (PMC10703444; doi:10.7554/eLife.88268)
Supplement: Supplementary file 3. — Relative difference < 0.3 is shown in bold letters and denotes parameters that reached convergence after 100,000 cycles using a burn-in of 250 and sampling every two cycles. [file elife-88268-supp3.docx]

**Supplemental File 3**

| Parameter | Effective Size | Relative difference |
| --- | --- | --- |
| **Loglik** | **7** | **0.16** |
| **Length** | **12** | **0.30** |
| **Sigma** | **21** | **0.16** |
| **Mu** | **37** | **0.09** |
| **Meanrates** | **19** | **0.16** |
| Scale | 19 | 0.57 |
| Alpha | 26 | 0.92 |
| **Nmode** | **70** | **0.29** |
| **Stat** | **8** | **0.37** |
| Statalpha | 20 | 0.94 |
| **Kappa** | **362** | **0.13** |
| Allocent | 10 | 0.51 |

Number of cycles = 92412; burn-in = 250; sampling = 2 cycles; 4 chains
